# Cough Syrup

**Published:** 1890-07

**Authors:** 


### COUGH SYRUP.

The following is said to be an excellent remedy for convulsive coughs:

| | |
|---|---|
| Sodium benzoate, | 5 parts. |
| Mint water | 40 parts. |
| Distilled water | 40 parts. |
| Syrup of orange peel | 10 parts. |

Mix. Dose, a teaspoonful may be taken whenever necessary. —*N. E. Med. Mo.*

---

A bushel of corn makes four gallons of whiskey. It sells for $16.00 at retail. The Government gets $3.60, the farmer 40 cents, the railroad $1.00, the manufacturers $4.00, the vender $7.00, and the drinker all that is left—delirium tremens.

---

Woodbury says that ten grains of the bicarbonate of soda in a half-ounce of an infusion of ura ursi every two hours will relieve acute inflammation of the bladder immediately.

